# The role of marine sediment diagenesis in the modern oceanic magnesium cycle

**DOI:** 10.1038/s41467-019-12322-2

**Published:** 2019-09-25

**Authors:** Richard D. Berg, Evan A. Solomon, Fang-Zhen Teng

**Affiliations:** 1 School of Oceanography, University of Washington, Seattle, WA 98195 USA; 2 Department of Earth and Space Sciences, University of Washington, Seattle, WA 98195 USA

**Keywords:** Element cycles, Marine chemistry

## Abstract

The oceanic magnesium cycle is largely controlled by continental weathering and marine authigenic mineral formation, which are intimately linked to long-term climate. Uncertainties in the magnesium cycle propagate into other chemical budgets, and into interpretations of paleo-oceanographic reconstructions of seawater δ^26^Mg and Mg/Ca ratios. Here, we produce a detailed global map of the flux of dissolved magnesium from the ocean into deeper marine sediments (greater than ∼1 meter below seafloor), and quantify the global flux and associated isotopic fractionation. We find that this flux accounts for 15–20% of the output of magnesium from the ocean, with a flux-weighted fractionation factor of ∼0.9994 acting to increase the magnesium isotopic ratio in the ocean. Our analysis provides the best constraints to date on the sources and sinks that define the oceanic magnesium cycle, including new constraints on the output flux of magnesium and isotopic fractionation during low-temperature ridge flank hydrothermal circulation.

## Abstract (web summary)

The oceanic magnesium cycle is closely linked to Earth’s carbon cycle and long-term climate change, due to processes such as continental weathering and authigenic mineral formation. Here, the authors update the global oceanic magnesium budget by quantifying the flux of magnesium from oceans to marine sediments and the associated isotopic fractionation.

## Introduction

The oceanic magnesium cycle is primarily a balance between weathering on land, high- and low-temperature hydrothermal alteration of the basaltic oceanic lithosphere, and formation of sedimentary carbonates and aluminosilicates^1–17^. Because these processes are also major drivers of long-term carbon dynamics and affect many other element cycles in the ocean, paleo-oceanographic reconstructions of the magnesium cycle can provide information about long-term climate and element cycling in the ocean. Fluctuations in Mg/Ca ratios of biogenic sedimentary carbonates reflect oscillations between “hot-house” and “ice-house” conditions, and the evolution of magnesium isotope ratios in the ocean have been interpreted as recording changes in silicate weathering on land and in the oceanic crust^10,12,17,18^. Models that use paleo-oceanographic reconstructions of proxies such as Mg/Ca or δ^26^Mg rely on knowledge of the modern-day magnesium cycle to use as a benchmark for quantification of the changes in the past. However, large uncertainties in the modern-day magnitudes propagate uncertainty into interpretations of past changes. By better defining the magnitudes of the current input and output processes of magnesium in the modern ocean, the changes in the sources and sinks through geologic time can be more accurately included in models that explore the dynamics of the Mg/Ca ratio and δ^26^Mg in the ocean.

Magnesium interacts with major geochemical cycles, such as the carbon and calcium cycles during the formation of authigenic carbonates and aluminosilicates^14^. Authigenic carbonate precipitation tends to occur to a greater extent at continental margins, where the burial of organic matter is greatest, and rates of alkalinity production are higher from organic matter degradation, sulfate reduction, and anaerobic oxidation of methane. Magnesium is incorporated into the crystal structure of these authigenic carbonates as they form in situ within or below the sulfate reduction zone, in the form of low- and high-magnesium carbonates. Authigenic carbonates often form with relatively high magnesium contents due to the high Mg/Ca ratio and low inhibitory sulfate concentrations in the pore water, particularly deeper within the sulfate reduction zone or at the sulfate-methane transition zone^18–22^. In addition to precipitation of primary authigenic carbonate, magnesium may be depleted in pore waters during the recrystallization of low-magnesium biogenic carbonate to forms of carbonate that have a greater magnesium content than the original biogenic calcite^11,18,23,24^. Authigenic aluminosilicate formation is also a potentially important sink for magnesium in marine sediments, where it can be incorporated into the authigenic phase in a greater stoichiometric ratio than in the original primary silicate from which it formed, resulting in the release of calcium and other cations to pore waters^25^. Many authigenic aluminosilicate formation reactions occur in marine sediments, but in all of these reactions, the products are primarily cation-rich clays with high surface areas and high cation exchange capacities^25–29^. Formation of these minerals typically results in the net uptake of magnesium from pore waters, with the magnitude of uptake dependent on the specific mineral or glass compositions involved in the reaction. Because these mineral formation and alteration reactions involve carbon and other major elements, variations in the amount of authigenic carbonate and aluminosilicate formation through time affect the long-term carbon cycle and other element cycles.

The flux of dissolved magnesium into marine sediments from the overlying ocean is widely driven by molecular diffusion that occurs as pore water magnesium is depleted during authigenic mineral formation in the sediment column, as well as the direct burial of seawater as pore water that results from sediment accumulation on the seafloor. This global flux of magnesium into marine sediments has been demonstrated to be a potentially important part of the oceanic magnesium cycle^30–32^. Depletion of magnesium in marine pore waters is nearly ubiquitously observed in pore water concentration profiles from scientific ocean drilling, exhibiting a clear sedimentary sink for magnesium meters to 10’s of meters below the seafloor, particularly in continental margin environments. The formation of authigenic minerals within the sediment column creates a diffusional gradient that drives the transfer of dissolved magnesium from the more concentrated ocean into the depleted pore waters. Pore water concentration profiles from most environments exhibit no net release of magnesium at greater depths, indicating a stable sedimentary sink for magnesium. However, in some localized regions of the ocean, other processes may cause magnesium to flux from pore waters into the overlying ocean, such as high-magnesium mineral dissolution or diffusion from relict brine and evaporite deposits^27,33^.

Depletion of magnesium in the pore waters is accompanied by variations in the isotopic ratio of ^26^Mg/^24^Mg. These isotopic variations reflect the balance between formation of isotopically-light carbonates and isotopically-heavy aluminosilicates in the sediment column. Measured fractionations of magnesium isotopes in marine pore waters are large enough to significantly affect the oceanic magnesium isotope ratio over time periods of millions of years through exchange with the overlying ocean^10,11,34^. With authigenic mineral formation reactions fractionating the pore water isotopes, the ratio of the diffusional gradients of the individual isotopes will vary from their concentration ratio in the overlying ocean, and thus the diffusion results in removal of magnesium with a different isotopic ratio than the overlying oceanic source. This diffusional fractionation is dampened by the direct burial of seawater as pore water, which is buried with the same isotopic ratio as the overlying ocean, as sediment accumulates on the seafloor. The burial velocity of pore water varies with sediment accumulation rate and compaction regimes, but is always downward with respect to the seafloor in systems with steady-state compaction^35,36^. After undergoing alteration during sediment diagenesis, much of the chemically and isotopically altered pore water eventually returns to the ocean in its altered form during compaction at subduction zones and other compressive tectonic regimes.

Here, we calculate the magnitude and distribution of magnesium fluxes into marine sediments from the overlying ocean using available scientific ocean drilling data from 1968–2015. Fluxes are calculated considering both molecular diffusion and burial of seawater as pore water at 269 ocean drilling sites from a wide-variety of continental margin and abyssal environments. We find that the flux of dissolved magnesium associated with diffusion and pore water burial accounts for 15–20% of the total output of magnesium from the ocean, typically with higher fluxes near the continental margins and lower fluxes in the abyssal ocean. We also find that the isotopic fractionation associated with the flux of magnesium from the ocean into marine sediments is slightly negative, acting to increase the ^26^Mg/^24^Mg ratio in the ocean. Using these new findings, we better constrain the global oceanic magnesium budget, including the low-temperature ridge-flank hydrothermal sink.

## Results

### Dissolved magnesium fluxes at individual locations

**Fig. 1:**
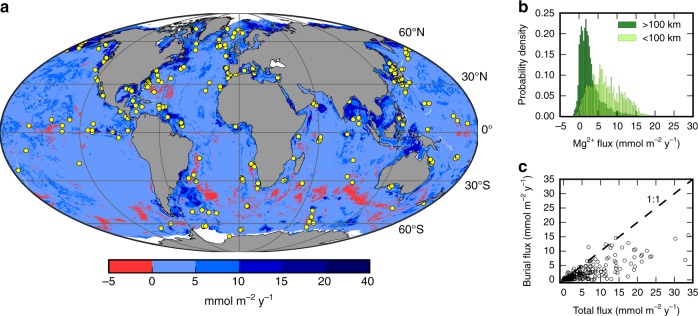
Global magnesium fluxes into marine sediments. **a** Global distribution of net magnesium fluxes into marine sediments (millimoles per square meter per year); positive values are from the ocean into the seafloor; dots are the individual ocean drilling sites with measured fluxes. **b** Histogram showing the distribution of net magnesium fluxes near (<100 km, *n* = 396,962) and farther away (>100 km, *n* = 4,8811,049) from the continental margins. **c** Comparison of the magnitude of the net magnesium fluxes (*n* = 269) at each location versus the flux associated with pore water burial. Source data are provided as Source Data files

Total fluxes at the 269 sites (Fig. 1) range from −0.03 to 33.8 mmol m^−2^ y^−1^, with a median value of 2.6 mmol m^−2^ y^−1^. Positive values represent a flux from the ocean into the seafloor. Monte Carlo simulation is conducted to estimate the total uncertainty of the flux at each site, with a median uncertainty of 0.9 mmol m^−2^ y^−1^. Pore water concentration gradients near the sediment-water interface (centimeters to meters) do not typically exhibit significant differences from the deeper gradients at depths of meters to 10’s of meters in non-advective systems^20,37–39^. Thus, the concentration gradients calculated from ocean drilling data are likely representative of gradients across the sediment-water interface, although greater fluxes may be driven by additional uptake in the top millimeters to centimeters of the sediment column that are not quantified here. The magnesium flux associated with pore water burial is a significant component of the total magnesium flux at most locations (Fig. 1c).

### Global dissolved magnesium flux into marine sediments

Due to the sparse spatial coverage of ocean drilling sites in relation to the variability of magnesium fluxes into the seafloor, interpolation procedures are not able to consistently capture commonly-observed variations such as the higher-magnitude fluxes closer to the continental margins. However, the ocean drilling dataset does represent a wide variety of environments, including abyssal ocean basins, convergent and divergent margins, and back-arc basins. More importantly, the dataset represents a wide range of areas with differing sediment accumulation rates, organic carbon sources and fluxes, temperature regimes, and lithologies. For this reason, the 269 magnesium fluxes are used as a training dataset for modeling the global distribution of fluxes using gradient-boosting regression (GBR), a supervised machine learning technique. The characteristic diversity of the dataset lends itself well to prediction of fluxes in the global ocean from globally-gridded datasets of other variables such as sediment accumulation rate, surface sediment porosity, surface ocean productivity, bottom water temperature, and water depth (Table 1). Similar methods have previously been used to estimate the global distribution of sulfate reduction rates, as well as gas hydrate occurrence and surface sediment porosity^40–42^. Because localized processes, such as seafloor seeps and diffusion from relict brines, are not reflected in available globally-gridded datasets, the regression technique would not account for their effect on the distribution of fluxes and so sites affected by these processes are not included in the regression. However, these areas are much more limited in extent and magnitude than the biogeochemically-driven fluxes of magnesium into marine sediments^23,30^. In addition, modern-day carbonate formation and dolomitization taking place at platform carbonate sites with large-scale advection in the upper sediment section are not included in this analysis due to the uncertainties in advection velocities.

**Table 1:** Globally-gridded predictor datasets

Dataset	Reference	Source	Original grid resolution
Global relief (water depth)	56	https://www.ngdc.noaa.gov/mgg/global/global.html	1 min
Surface sediment porosity	41	https://agupubs.onlinelibrary.wiley.com/doi/full/10.1002/2015GL065279	5 min
Surface productivity	41	https://agupubs.onlinelibrary.wiley.com/doi/full/10.1002/2015GL065279	5 min
Sediment accumulation rate	53–57	Calculated from sediment thickness and crustal ages	5 min
Bottom water temperature	51	https://www.nodc.noaa.gov/OC5/woa13/	0.25 deg

Datasets used in magnesium flux regression models, with online sources and spatial resolutions

The GBR results indicate that fluxes are generally higher near the continental margins and lower in the abyssal ocean basins (Fig. 1a, b). The higher fluxes of magnesium into sediments near continental margins are consistent with what is expected in areas with higher organic carbon burial rates, alkalinity production, and authigenic carbonate precipitation. The total flux of magnesium from the ocean into marine sediments calculated using the GBR method is 1.1 ± 0.5 Tmol y^−1^ (1 Tmol = 10^12^ mol), a similar magnitude as the high-temperature hydrothermal flux^7^. We also calculate the total flux using random forest regression and multiple linear regression, for comparison. The flux calculated with the GBR method is the same as that calculated using random forest regression (1.1 Tmol y^−1^), and similar to the multiple linear regression result (1.3 Tmol y^−1^), with the GBR method having the greatest accuracy of the three methods (See Methods sections for details). This value is also within the 0.9–1.8 Tmol y^−1^ range of the global diffusive flux of magnesium estimated using an interpolation procedure^43^.

### Isotopic fractionation associated with magnesium fluxes

**Fig. 2:**
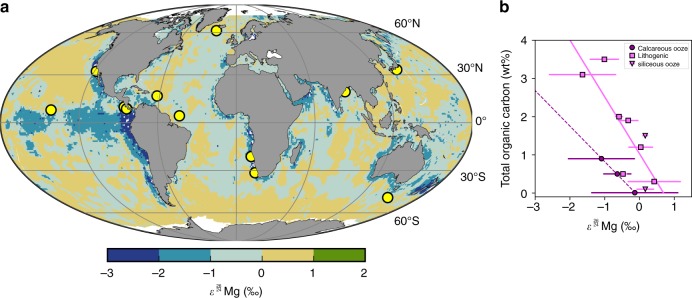
Fractionation of magnesium isotopes into marine sediments. **a** Global distribution of the isotopic fractionation of magnesium isotopes associated with the magnesium flux into the marine sediment column. Yellow dots are the thirteen ocean drilling locations where the fractionations are calculated. **b** Isotopic fractionation of magnesium isotopes plotted versus total organic carbon in the upper sediment column. Linear regressions are fit to the data for calcareous sediments (dark purple, *n* = 3, *R*^2^ = 0.9996, *p* < 0.05), and siliceous sediments (light purple, *n* = 10, *R*^2^ = 0.8171, *p* < 0.05) separately. Circles are calcareous oozes, squares are lithogenic sediments, and triangles are siliceous oozes. Epsilon values are for fractionation from the ocean bottom water into the seafloor. Error bars represent the 1σ standard deviation of the epsilon values. Gridded source data for Fig. 1a are provided as a Source Data file

In addition to the global flux distribution, we calculate the net fractionation associated with the magnesium flux into the sediment column at thirteen ocean drilling locations (Fig. 2). The thirteen locations include sites from continental margins and abyssal ocean basins, with a range of lithologies and organic carbon burial rates. Authigenic mineral formation reactions occurring in the sediment column change the isotopic ratios of the pore water magnesium, creating an isotopic gradient between the overlying ocean water and the pore waters. This isotopic gradient results in a fractionation associated with the diffusional transport of magnesium from the ocean into the pore waters of the sediments. In contrast, the burial of seawater during sediment accumulation occurs without isotopic fractionation. So, while pore water burial increases the total flux of magnesium into the sediment column, it acts to dampen the magnitude of the net fractionation factor associated with that flux of magnesium.

The fractionations associated with the fluxes from the ocean into the sediment column range from −1.6‰ to 0.4‰, within the range for authigenic carbonate formation and aluminosilicate formation, respectively. We find that the net flux into the modern seafloor acts to increase the magnesium isotopic ratio in the ocean, reflecting greater fractionation due to authigenic carbonate formation compared to aluminosilicate formation in the upper sediment column at most sites. There is no clear correlation between the magnitude of the fluxes and the isotopic fractionation factors. Rather, sites with more positive fractionations tend to be those with clay-dominated lithologies and low organic carbon contents, while sites with greater amounts of organic carbon or more carbonate-dominated sediments have more negative fractionation values (Fig. 2b). These findings are consistent with studies of net fractionation of magnesium isotopes during diagenesis^24,34^. A simple binned regression of sites (Fig. 2b) based on total organic carbon content and lithology of surface sediments provides a global distribution of the isotopic fractionation associated with the global flux of magnesium into marine sediments (Fig. 2a). From this regression, we calculate a flux-weighted isotopic fractionation factor of 0.9994 associated with the global flux, indicating the dominance of authigenic carbonate formation in driving the fractionation associated with the global diffusional magnesium flux into marine sediments (see Table 2). These results are also consistent with the slight enrichment in light magnesium isotopes of global subducting sediments compared with average continental crust^44^.

**Table 2:** Ocean-sediment magnesium fractionation model parameters

Leg/ Expedition	Site	Holes	Number of datapoints used for gradients	Fit type	Epsilon (‰)	Standard deviation	Pore water δ^26^Mg data source	Total organic carbon in upper sediment column (wt%)	Dominant lithology in upper sediment column
170	1039	BC	4	Linear	0.18	0.09	This study	1.5	Siliceous ooze
315	C0002	BD	3	Linear	−0.77	0.19	This study	0.5^a^	Lithogenic clay
344	U1414	A	3	Linear	−0.33	0.30	This study	1.9	Lithogenic clay
334	U1378	B	5	Linear	−0.52	0.14	This study, 63	2	Lithogenic clay
NGHP01	18	A	3	Linear	0.03	0.36	This study	1.1^a^	Lithogenic clay
189	1171	ACD	6	Linear	−0.64	0.41	24	0.5	Calcareous ooze
162	984	ABCD	3	Linear	0.41	0.77	34	0.3	Lithogenic clay
167	1012	A	3	Linear	−1.6	0.97	34	3.1	Lithogenic clay
154	925	ABE	3	Linear	−0.10	1.3	34	0.01	Calcareous ooze
175	1082	A	4	Linear	−1.0	0.42	34	3.5	Lithogenic clay
175	1086	A	4	Linear	−1.1	0.96	34	0.9	Calcareous ooze
199	1219	AB	3	Linear	0.17	0.25	34	0.1	Siliceous ooze
340	U1395	B	4	Linear	0.43	0.34	63	0.6	Biovolcaniclastic/ hemipelagic

^a^Total organic carbon concentrations in the upper sediment column at IODP Site C0002 and NGHP01 Site 18 were estimated based on the concentrations at nearby IODP Site C0001 and NGHP01 Site 19, respectively. NGHP01 refers to the Indian National Gas Hydrate Program Expedition 01

## Discussion

By quantifying this output of magnesium and associated isotopic fractionation, we can more accurately constrain the modern global magnesium budget, including the low-temperature ridge flank sink. The dominant sources of magnesium to the ocean are rivers and groundwater, which input a combined total of about 7.6 Tmol y^−1^, with more minor contributions from subduction zone reflux and weathering of seafloor peridotites (Table 3). Assuming the present-day oceanic magnesium cycle is in steady state, the sinks of high and low temperature hydrothermal circulation, marine sediments, and preserved biogenic carbonates also have a combined magnitude of about 7.6 Tmol y^−1^. See the Methods section for mass balance calculations and data sources. After accounting for independent estimates of the magnesium fluxes and isotopic fractionations associated with the known sources and sinks of oceanic magnesium, including the flux into the marine sediment column found in this study, low-temperature ridge flank hydrothermal circulation accounts for 4.3 Tmol y^−1^ of the output flux, making it the largest sink for magnesium in the ocean (Table 3).

**Table 3:** Global oceanic magnesium budget

Input processes	Flux (Tmol y^−1^)	δ^26^Mg (‰)	References
Rivers	5.2 (4.8–7.1)	−1.09 (−1.14 to −1.04)	1, 2
Groundwater	1.8 (N.P.)	−1.2 (1σ = 0.2)	3, 4, 72–76
Seafloor peridotite weathering	0.15 (0.0000018–4.1)	−1.31 (N.P.)	5, 6
Subduction zone reflux	0.43 (N.P.)	−0.52 (1σ = 0.54)	11, 34, 51, this study
**Output processes**			
Flux into marine sediment column	1.1 (0.6–1.6)	−1.4	This study
High-temperature ridge crest circulation	1.5 (0.53–2.1)	−0.83	7
Low-temperature ridge flank circulation	4.3	−0.76	This study, calculated from mass balance
Biogenic carbonate preservation	0.6 (N.P.)	−3.5 (N.P.)	8–10, 13, 16, 17
Ion adsorption onto detrital clays	0.1 (0–0.2)	−0.83	14, 15

The steady-state budget includes the inputs of magnesium to the ocean and output processes of magnesium from the ocean, with the corresponding isotopic delta values of the sources or sinks. Values are those given in the respective references for each input/output process, with ranges or standard deviations given, where available, in parentheses (N.P. = not provided in the referenced literature). Uncertainty estimates are not made for those values calculated from mass balance, or that are based on the isotopic composition of seawater. See Methods for further discussion of the values included in the budget. For reference, the magnesium isotopic value of seawater is −0.83‰ 1σ = 0.034‰^60^

Using the fluxes and isotopic values in Table 3, the isotopic composition of the low-temperature ridge flank sink is found to be slightly heavier than seawater, indicating a slightly positive net isotopic fractionation (see Methods for mass balance calculation and assumptions). The small net fractionation of 1.00007 associated with the global low-temperature ridge flank sink is contrasted with the fluid-basalt fractionation factor of 1.00055 calculated from carbonate-barren basaltic basement at IODP Site 1253 (Tables 4 and 5), which is likely dominated by high-magnesium clay formation as found at IODP Site 1256 near the East Pacific Rise, and the carbonate-poor lower basement of Site 801C outboard of the Mariana Trench^45,46^. The low net fractionation value could be explained by the observation that carbonate veins commonly form during low-temperature hydrothermal circulation, as has been observed in magnesium isotope measurements on altered basalts from ODP Site 504B near the Costa Rica Rift and the carbonate-rich upper basement at Site 801C^46,47^. Precipitation of isotopically-light carbonate during low-temperature hydrothermal circulation can reduce the net fractionation associated with this sink, counteracting the fractionation caused by formation of isotopically-heavy aluminosilicates.

**Table 4:** Mg isotope values of IODP Site 1253 basaltic basement fluid

Expedition	Site	Sample	δ^26^Mg (‰)	δ^26^Mg 2σ (‰)	δ^25^Mg (‰)	δ^25^Mg 2σ (‰)
301T	1253	MKG150	−1.22	0.04	−0.63	0.04
301T	1253	MKG140	−1.25	0.04	−0.63	0.04
301T	1253	MKG130	−1.28	0.05	−0.65	0.05
301T	1253	MKG120	−1.27	0.05	−0.65	0.05

**Table 5:** Rayleigh fractionation calculation parameters

Sample	*R*	*R*_0_	*X* (mM)	*X*_0_ (mM)	*α*	*ε* (‰)
MKG150	0.139620	0.139674	25.0	54.0	1.000502	0.50
MKG140	0.139615	0.139674	24.1	54.0	1.000521	0.52
MKG130	0.139611	0.139674	25.4	54.0	1.000599	0.60
MKG120	0.139612	0.139674	25.3	54.0	1.000587	0.59

Values used in the calculation of fractionation factor associated with basalt alteration, from IODP Site 1253 CORK samples^62^

Sparse proxy data for the magnesium concentrations in the ocean for the past ∼15 My suggest that the magnesium budget of the ocean may be out of steady-state, with the inputs of magnesium greater by about 1 Tmol y^−1^ than the outputs^10,48^. The non-steady-state budget scenario, presented in Supplementary Table 1, would require the low-temperature ridge flank magnesium sink to have an isotopic composition lighter than seawater, with a fractionation factor of 0.99985. Because authigenic clay formation is thought to dominate the magnesium uptake in these low-temperature hydrothermal systems, the non-steady state budget would suggest that there is another unquantified process that is either a source of heavier magnesium isotopes to the ocean, or a sink of lighter isotopes from the ocean.

A small net fractionation either positive (for steady-state mass balance) or negative (for non-steady state mass balance) during low-temperature hydrothermal circulation suggests that the marine δ^26^Mg record is primarily controlled by variations in continental weathering, biogenic carbonate formation, and particulate organic carbon burial that drives diagenetic processes in marine sediment. Additional factors that may be important controls on the δ^26^Mg record through time include the relative preservation rates of foraminifera tests versus coccoliths, and the relative amount of carbonate precipitation versus clay formation during low-temperature ridge flank circulation. These new constraints on the oceanic magnesium cycle offer greater insight into the present-day magnesium cycle and provide a benchmark for models and interpretations of the paleo-oceanographic record.

## Methods

### Data sources

Fluxes of magnesium into marine sediments were calculated using available data from the National Geophysical Data Center (https://www.ngdc.noaa.gov/mgg/geology/dsdp), Janus (http://www-odp.tamu.edu/database), LIMS (http://web.iodp.tamu.edu/LORE), and J-CORES (http://sio7.jamstec.go.jp) databases that house drilling data from the Deep Sea Drilling Project (DSDP), ODP, and IODP. Additional data were compiled from the Indian National Gas Hydrate Program (NGHP) Expedition 01^49^. This compiled dataset includes data collected with both the JOIDES Resolution and D/V CHIKYU. Only sites with high-quality data were used, limiting the sites to those with at least three pore fluid magnesium concentration measurements in the upper sediment column that form a gradient to the ocean bottom-water concentration at the sediment-water interface, and are not affected by unquantified advection of pore water, brine diffusion, or sample collection artifacts due to gas hydrate dissociation.

### Solute flux calculations

Fluxes at individual sites are directly calculated using measurements of concentration gradients, porosity-depth profiles, sediment accumulation rates, and in situ temperatures. This approach directly calculates the combined effect of molecular diffusion and pore water burial using the 1-dimensional advection diffusion equation in porous media:

$$J = - \varphi _0D_{\mathrm{s}}\frac{{dC}}{{dz}} + b_0C_0$$

where *J* is the flux into the sediment column. *φ*_0_ is the porosity of the surface sediment found using the best fit of Athy’s Law to the measured porosity profile. *dC/dz* is the concentration gradient (mol m^−4^) at the sediment-water interface found using an exponential fit to the uppermost four or more measurements. *C*_0_ is the magnesium concentration (mol m^−3^) at the sediment-water interface. *D*_s_ is the effective sedimentary diffusion coefficient (m^2^ y^−1^), accounting for sediment tortuosity with the relationship^50^: *D*_s_ = *D*_sw_/(1−ln(*φ*^2^)), where *D*_sw_ is the molecular diffusion coefficient in sea water corrected for bottom water temperatures from the World Ocean Atlas using the Stokes-Einstein equation^51^.

The term *b*_0_ is the volumetric pore water burial flux (m^3^ y^−1^) accounting for sediment compaction using the relationship:

$$b_0 = \frac{{\varphi _{\mathrm{L}}\left( {1 - \varphi _0} \right)}}{{\left( {1 - \varphi _{\mathrm{L}}} \right)}}s$$

where *φ*_L_ is the sediment porosity at depth where compaction of the sediment column becomes negligible, and **s** is the sediment accumulation rate found using a piecewise linear regression of the biostratigraphic data for each site. Monte Carlo simulation was used to estimate the standard deviations of each net magnesium flux based on the known uncertainty in the concentrations, porosities, and pore water burial rates.

### Regression model

Net magnesium fluxes at individual sites were used as a training dataset to predict the global distribution of magnesium fluxes into marine sediments to depths over about 1 meter below seafloor using the gradient-boosting regression technique in the scikit-learn Python package^52^. The supervised machine learning algorithm fits a decision tree model to the training data, and then sequentially fits a new decision tree to the residuals from the prior model fit for a specified number of iterations. The branching of the decision tree is based on the splits in other environmental parameters, or features, that effect the greatest reduction in variance in the training data associated with each branch. The models are then combined to find the best possible multidimensional fit of the training data to the other environmental parameters. Globally-gridded datasets of those environmental parameters are then used to predict the global distribution of magnesium fluxes.

The globally-gridded features used in the regression models are listed in Table 1. The data are all resampled to 5-min grid-registered grids using the Rasterio Python package. For sediment accumulation rate, long-term average sediment accumulation rate is calculated, as in Burwitcz et al., (2011)^53^, where sediment thickness in each grid cell is divided by the underlying crustal age. For sediment thicknesses, the Whittaker (2013)^54^ dataset is used where available, with the Laske and Masters^55–57^ dataset being used where needed.

### Regression model parameters and cross-validation

**Fig. 3:**
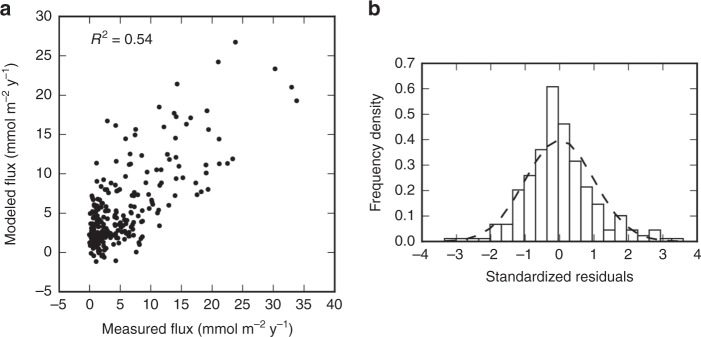
Gradient boosted regression results. **a** Measured magnesium flux values vs. model results via leave-one-out cross-validation (*R*^2^ = 0.54 and *p* < 0.01), using the gradient boosting regression technique with the parameters listed in Table 6. **b** Histogram of standardized residuals of the cross-validation (*n* = 269), with the dashed line showing an idealized normal distribution (mean = 0, standard deviation = 1)

Cross-validation is used to evaluate the accuracy of the regression, and to compare it to other potential methods. See Table 6 and Supplementary Tables 2 and 3 for the model parameters and Fig. 3 and Supplementary Figs. 1 and 2 for the cross-validation analyses. Three regression methods were compared using Leave-One-Out cross-validation to determine which method was best suited to the ocean drilling dataset. While the gradient-boosting regressor and random forest techniques provide similar predictive power, the gradient-boosting regression technique provides both the best predictive power, and is the most robust option to avoid over-fitting the model to training data, as indicated by the greater number of samples per leaf. The best-fit parameters for each method are listed in Table 6 and Supplementary Tables 2 and 3, along with the results of the cross-validation analysis. For gradient-boosting regression and random forest, the feature importances are listed, which are a measure of how much each feature reduces the variance of the model fit. For the multiple linear regression technique, the linear coefficients are listed.

**Table 6:** Gradient boosting regression parameters and results

*Model parameters*	
Loss function	least squares regression
Boosting stages	2000
Learning rate	0.01
Minimum samples per leaf	8
Quality of split criterion	Friedman mean-squared error
*Feature importances*	
Sediment accumulation rate	0.363
Surface sediment porosity	0.216
Bottom water temperature	0.144
Water depth	0.112
Surface ocean productivity	0.165
*Results*	
Coefficient of determination	0.542
Global Mg flux (Tmol y^−1^)	1.10

### Pore water magnesium isotope measurements

Pore water magnesium isotopes from ODP Site 1039 and 1040, and IODP Sites U1378/U1380, U1414, and C0002, and NGHP Site 18 were measured via multi-collector inductively-coupled plasma mass spectrometry (MC-ICPMS) in the Isotope Laboratory at the University of Washington. Sample preparation and column chemistry were conducted in a clean lab, and the procedures followed those of the previous studies^6,58^. The pore water samples were dried, and then re-dissolved in 1 N HNO_3_ before chromatographic separation. Cation exchange chromatography, using Bio-Rad AG50W-X8 (200–400 mesh) resin in 1 N HNO_3_, was performed twice on each sample to chemically separate the magnesium from other ions in the samples. Magnesium isotopic compositions were analyzed using the sample-standard bracketing method on a Nu Plasma II MC-ICPMS^59^. A seawater standard at variable concentrations was also analyzed with each batch of samples to monitor accuracy and reproducibility^60^. Magnesium isotopic data are reported in delta (δ) notation in per mil relative to DSM3 standard^61^. Repeated analyses indicate data reproducibility is ± 0.06‰ (2σ) or better for δ^26^Mg, far below the natural variations observed in the pore water profiles. Hydrothermal fluids from IODP Site 1253 CORK observatory samples were measured using the same procedures as the pore water samples^62^. All measured magnesium isotope analytical data are provided in the Supplementary Table 4. Additional pore water magnesium isotope values from ODP Sites 925, 984, 1012, 1082, 1086, 1171, and 1219, and IODP Sites U1378 and U1395 were obtained from published sources^24,34,63^. Other sites with available pore water magnesium isotope data are excluded that are either sparse near the sediment-water interface, affected by brine diffusion, or associated with unquantified pore water advection in the upper sediment section, including ODP Sites 807, 1003, 1052, 1196, 1265, and IODP Site U1403^17,63,64^. Values of δ^25^Mg are calculated using the mass-dependent relationship δ^25^Mg=0.516×δ^26^Mg for sites 925, 984, 1012, 1082, 1086, and 1219, for which δ^25^Mg values are not published^65,66^.

### Magnesium isotope fractionation calculations

To calculate concentrations of individual magnesium isotopes in pore waters, the absolute isotopic ratios are first calculated by:

$$\frac{{{\,}^{26}{\mathrm{{Mg}}}}}{{{\,}^{24}{\mathrm{{Mg}}}}}_{{\mathrm{{samp}}}} = \left( {\frac{{\delta ^{26}{\mathrm{{Mg}}}}}{{1000}} + 1} \right) \times \frac{{{\,}^{26}{\mathrm{{Mg}}}}}{{{\,}^{24}{\mathrm{{Mg}}}}}_{\mathrm{{DSM3}}}$$

$$\frac{{{\,}^{25}{\mathrm{{Mg}}}}}{{{\,}^{24}{\mathrm{{Mg}}}}}_{{\mathrm{samp}}} = \left( {\frac{{\delta^{25}{{\mathrm{{Mg}}}}}}{{1000}} + 1} \right) \times \frac{{{\,}^{25}{\mathrm{{Mg}}}}}{{{\,}^{24}{\mathrm{{Mg}}}}}_{{\mathrm{DSM}}3}$$

where ^26^Mg/^24^Mg_DSM3_ = 0.13979, and ^25^Mg/^24^Mg_DSM3_ = 0.12685^65^. Concentrations of individual magnesium isotopes (^26^Mg and ^24^Mg) in pore waters are calculated by:

$$\left[{{\,}^{24}{\mathrm{{Mg}}}} \right] = \frac{{\left[ {{\mathrm{{Mg}}}} \right]}}{{\frac{{{\,}^{26}{\mathrm{{Mg}}}}}{{{\,}^{24}{\mathrm{{Mg}}}}} + \frac{{{\,}^{25}{\mathrm{{Mg}}}}}{{{\,}^{24}{\mathrm{{Mg}}}}} + 1}}$$

$$\left[{{\,}^{26}{\mathrm{{Mg}}}} \right] = \left[{{\,}^{24}{\mathrm{{Mg}}}} \right] \times \frac{{{\,}^{26}{\mathrm{{Mg}}}}}{{{\,}^{24}{\mathrm{{Mg}}}}}$$

Fractionation factors associated with the fluxes of magnesium into the seafloor are then calculated by modeling the fluxes (*J*) of each isotope individually using the same model as for the bulk magnesium fluxes. These fluxes are then used to calculate the ocean-to-sediment-column fractionation factor (*α*) by:

$$\alpha = \frac{\frac{{J_{26}}}{{J_{24}}}}{\left( {\frac{{\,}^{26} \mathrm{Mg}}{{\,}^{24} \mathrm{Mg}}} \right)}_{\mathrm{O}}$$

where *J*_26_ and *J*_24_ are the fluxes of ^26^Mg and ^24^Mg into the seafloor, respectively, and (^26^Mg/^24^Mg)_O_ is the isotopic ratio in the ocean (0.13967)^60^. Monte Carlo simulation is used to estimate the standard deviations of each fractionation factor based on the known uncertainty in the isotopic ratios, porosities, and pore water burial rates.

### Site 1253 magnesium isotopes and fractionation calculation

The fractionation factors (*α*) associated with the transfer of magnesium from the IODP Site 1253 low-temperature hydrothermal fluid to the altered basalt are calculated as a Rayleigh fractionation:

$$\frac{R}{{R_0}} = \left( {\frac{X}{{X_0}}} \right)^{\alpha - 1}$$

where *R* is the ^26^Mg/^24^Mg_sample_, *R*_0_ is the ^26^Mg/^24^Mg_ocean_, *X* is the magnesium concentration in the sample (basaltic basement fluid), and *X*_0_ is the magnesium concentration in the ocean. Epsilon (*ε*) values are calculated from *α* by the relationship:

$$\varepsilon = \left( {\alpha - 1} \right) \times 1000$$

### Global extrapolation of magnesium isotope fractionation

The flux-weighted average ocean-sediment magnesium fractionation is calculated as:

$$\varepsilon _{{\mathrm{global}}} = \frac{{\mathop {\sum }\nolimits_{{\mathrm{i}} = 1}^n J_{\mathrm{i}} \times \varepsilon _{\mathrm{i}}}}{{J_{{\mathrm{global}}}}}$$

where *J* is the magnesium flux and *ε* is the ocean-sediment epsilon value, calculated at all *n* gridspaces. The *ε* value at each location is calculated separately for carbonate-dominated sediments and silicate-dominated sediments correlated to the total organic carbon (TOC) content of the top-most sediments at each location by the following equations:

For carbonate lithologies: *ε* = −1.47 × TOC – 0.11

For silicate lithologies: *ε* = −0.93 × TOC + 1.06

The lithology dataset of Dutkiewitcz et al., was used to globally determine the areas dominated by either carbonate or silicate sediments^67^. The silicate lithologies are defined as including gravel and coarser, sand, silt, clay, radiolarian ooze, diatom ooze, sponge spicules, ash and volcanic sand/gravel, and siliceous mud. The carbonate lithologies are defined as including calcareous ooze, mixed calcareous/siliceous ooze, shells, and coral fragments, and fine-grained calcareous sediment. The global dataset of Lee et al., 2019, was used to determine total organic carbon contents of the surface sediments^68^.

The global distribution of fractionation factors associated with the fluxes of magnesium into the seafloor is determined by linear regression of the fractionation factors with total organic carbon in the uppermost sediment column at each site. Regressions are done separately for sites dominated by silicate and carbonate lithologies in their uppermost sediment columns. The global distribution is then calculated by applying the regression relationships to globally-gridded datasets of surface sediment lithology and organic carbon content^67,68^.

### Oceanic magnesium budget ranges and mass-balance calculation

The sources of magnesium to the oceans include rivers (4.8–7.1 Tmol y^−1^)^1^, fresh submarine groundwater input (∼1.8 Tmol y^−1^)^3,69,70^, subduction zone reflux (∼0.43 Tmol y^−1^)^71^, and weathering of seafloor peridotites (0.0000018–4.1 Tmol y^−1^)^5^. Isotopic values for the rivers and weathering of seafloor peridotite are from published literature^2,6^.

The isotopic value for the reflux of magnesium into the ocean from compaction and dehydration reactions taking place at subduction zones (−0.52‰ ± 0.54‰, 1σ, *n* = 6) is estimated as the average isotopic composition of the deepest measured pore water magnesium isotopic composition from the six ocean drilling sites located on subducting plates or on convergent continental margins with measured values deeper than 200 mbsf (ODP Sites 807, 1039, 1040, 1219, and IODP Sites U1378 and U1414)^11,34^. The fluids at these depths represent the deep fluid that is expelled from convergent margins as sediments and pore space are tectonically compacted and minerals are dehydrated with increasing temperatures and pressures. This isotopic value is similar to the mean value of all magnesium isotope measurements from these locations at all depths (−0.62‰ ± 0.36‰, 1σ, *n* = 65).

The magnesium isotopic value for the groundwater source (−1.2‰ ± 0.2‰, 1σ, *n* = 27) is calculated as the mean value measured in silicate- and carbonate-dominated shallow groundwater reservoirs^4,72–76^. The mean isotopic value of groundwater in shallow silicate reservoirs (−1.28‰ ± 0.17‰, 1σ, *n* = 12) is within the 1σ error of the mean value in shallow carbonate reservoirs (−1.18‰ ± 0.17‰, 1σ, *n* = 15). Groundwater input from deeper reservoirs is likely much less than the shallow reservoir input into the ocean due to low-permeability confining layers restricting flow into the ocean^69,77^.

The output processes of magnesium from the ocean include the dissolved flux into the sediment column, high-temperature hydrothermal circulation^7^, low-temperature hydrothermal circulation, biogenic carbonate precipitation^78^, and ion adsorption onto detrital clays^14^. The flux into the seafloor and associated isotopic composition are from this study. The isotopic composition of the high-temperature sink is implied to be the same as seawater because it is fully depleted in high-temperature hydrothermal fluid. The low-temperature hydrothermal circulation flux and isotopic composition are calculated as described by the mass-balance equations below. The biogenic carbonate isotopic composition is estimated based on 50% of the flux due to formation of coccoliths, with isotopic values ranging from −1 to −3‰, and the other 50% of the flux due to formation of foraminifera tests, with isotopic values ranging from −4.2 to −5.5‰^9,10,13,16,17^. Ion adsorption onto clays has been observed to occur with no measurable fractionation relative to the seawater with which it is in equilibrium^15^.

The low-temperature hydrothermal circulation sink for magnesium is calculated using the other quantified fluxes of the steady-state oceanic magnesium cycle:

$$J_{{\mathrm{lthc}}} = J_{\mathrm{r}} + J_{{\mathrm{gw}}} + J_{{\mathrm{szr}}} + J_{{\mathrm{spw}}} - J_{{\mathrm{ms}}} - J_{{\mathrm{hthc}}} - J_{{\mathrm{bc}}} - J_{{\mathrm{ia}}}$$

where J_r_ is the river flux, *J*_gw_ is the groundwater flux, *J*_szr_ is the subduction zone reflux, *J*_spw_ is the seafloor peridotite weathering flux, *J*_ms_ is the marine sediment flux, *J*_hthc_ is the high-temperature hydrothermal circulation flux, *J*_bc_ is the biogenic carbonate flux, and *J*_ia_ is the ion adsorption flux.

For the non-steady-state magnesium budget presented in Supplementary Table 1, the low-temperature hydrothermal circulation sink for magnesium is calculated using the other quantified fluxes of the oceanic magnesium cycle, with sinks totaling 1 Tmol y^−1^ less than sources:

$$J_{{\mathrm{lthc}}} + 1 = J_{\mathrm{r}} + J_{{\mathrm{gw}}} + J_{{\mathrm{szr}}} + J_{{\mathrm{spw}}} - J_{{\mathrm{ms}}} - J_{{\mathrm{hthc}}} - J_{{\mathrm{bc}}} - J_{{\mathrm{ia}}}$$

where J_r_ is the river flux, *J*_gw_ is the groundwater flux, *J*_szr_ is the subduction zone reflux, *J*_spw_ is the seafloor peridotite weathering flux, *J*_ms_ is the marine sediment flux, *J*_hthc_ is the high-temperature hydrothermal circulation flux, *J*_bc_ is the biogenic carbonate flux, and *J*_ia_ is the ion adsorption flux. The isotopic mass balance is then calculated using the same method as the steady-state budget, described in the Methods section.

To calculate the isotopic composition of the of the low-temperature hydrothermal circulation sink, the following mass-balance is used:

$$\delta _{{\mathrm{lthc}}} = \frac{{\delta _{\mathrm{r}}J_{\mathrm{r}} + \delta _{{\mathrm{gw}}}J_{{\mathrm{gw}}} + \delta _{{\mathrm{szr}}}J_{{\mathrm{szr}}} + \delta _{{\mathrm{spw}}}J_{{\mathrm{spw}}} - \delta _{{\mathrm{ms}}}J_{{\mathrm{ms}}} - \delta _{{\mathrm{hthc}}}J_{{\mathrm{hthc}}} - \delta _{{\mathrm{bc}}}J_{{\mathrm{bc}}} - \delta _{{\mathrm{ia}}}J_{{\mathrm{ia}}}}}{{J_{{\mathrm{lthc}}}}}$$

where *δ*_r_ is the river δ^26^Mg value, *δ*_gw_ is the groundwater δ^26^Mg value, *δ*_szr_ is the subduction zone reflux δ^26^Mg value, *δ*_spw_ is the seafloor peridotite weathering δ^26^Mg value, *δ*_ms_ is the marine sediment δ^26^Mg value, *δ*_hthc_ is the high-temperature hydrothermal circulation δ^26^Mg value, *δ*_bc_ is the biogenic carbonate δ^26^Mg value, and *δ*_ia_ is the ion adsorption δ^26^Mg value.

## Supplementary information

Supplementary Information

Source Data

## Data Availability

The source data underlying Fig. 1a–c, Fig. 2a, and additional site metadata are provided as Source Data files. Correspondence and requests for materials should be addressed to R.D.B.

## Code availability

The Python code written for modeling of dissolved magnesium fluxes, fractionation factors, age-depth relationships, and porosity profiles at individual locations is available from https://github.com/rickdberg/solute_flux. In addition, all code, including that written for estimating the global magnesium fluxes, is available from R.D.B.
