# Supplementary material for: The role of marine sediment diagenesis in the modern oceanic magnesium cycle
Source: Nat Commun. 2019 Sep 25;10:4371. doi: 10.1038/s41467-019-12322-2 (PMC6761265; doi:10.1038/s41467-019-12322-2)
Supplement: Supplementary file 1 — Supplementary Information [file 41467_2019_12322_MOESM1_ESM.pdf]

Supplementary Information

The role of marine sediment diagenesis in the modern oceanic magnesium cycle

Berg et al.

Supplementary Figures

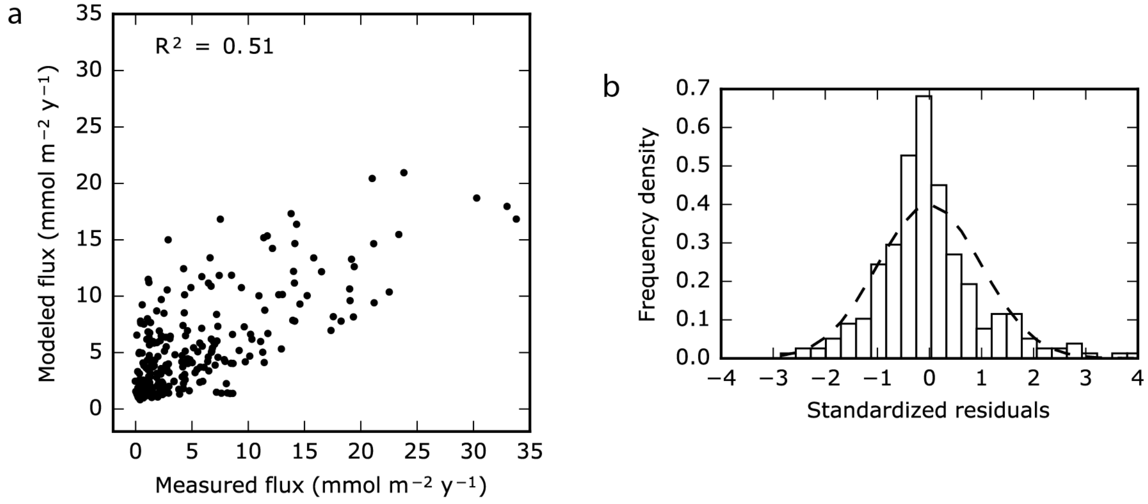

Supplementary Figure 1. A) Measured magnesium flux values vs. model results via leave-one-out cross-validation, using the random forest regression technique with the parameters listed in Supplementary Table 2. **B)** Histogram of standardized residuals of the cross-validation (n = 269), with the dashed line showing an idealized normal distribution (mean = 0, standard deviation = 1).

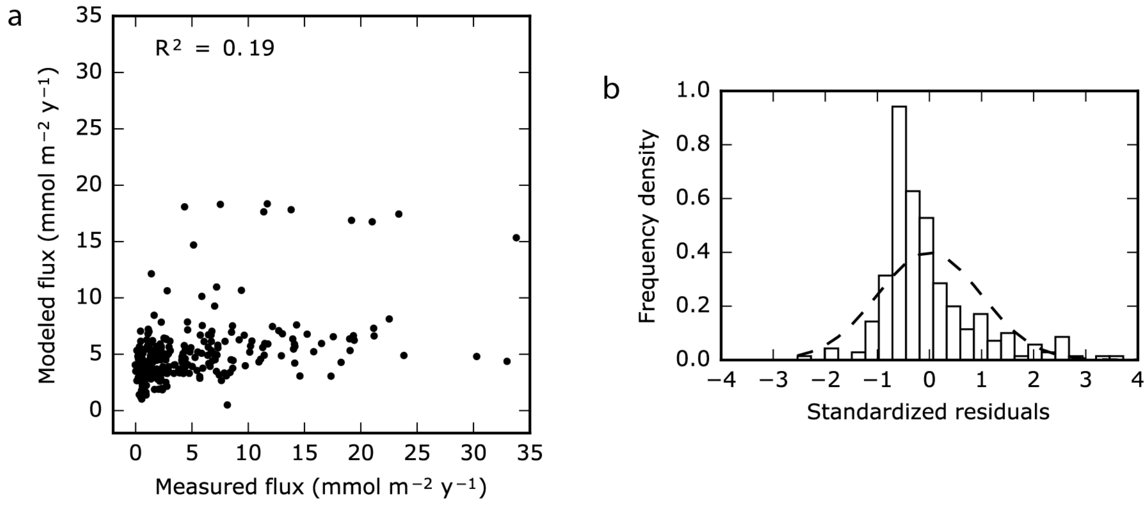

Supplementary Figure 2. A) Measured magnesium flux values vs. model results via leave-one-out cross-validation, using the multiple linear regression technique with the parameters listed in Supplementary Table 3. **B)** Histogram of standardized residuals of the cross-validation (n = 269), with the dashed line showing an idealized normal distribution (mean = 0, standard deviation = 1).

Supplementary Tables

Sources	Flux (Tmol y^{-1})	δ^{26}Mg (‰)
Rivers	5.2	-1.09
Groundwater	1.8	-1.2
Seafloor peridotite weathering	0.15	-1.31
Subduction zone reflux	0.43	-0.34
**Sinks**		
Marine sediments	1.1	-1.4
High-temperature ridge crest circulation	1.5	-0.83
Low-temperature ridge flank circulation	3.3	-0.98
Biogenic carbonates	0.6	-3.5
Ion adsorption onto detrital clays	0.1	-0.83

Supplementary Table 1. Non-steady state magnesium budget of the ocean, assuming 1 Tmol y^{-1} greater sources than sinks.

Model parameters	
Number of trees	200
Minimum samples per leaf	3
Quality of split criterion	Friedman mean-squared error
**Feature importances**	
Sedimentation rate	0.488
Water depth	0.148
Surface ocean productivity	0.124
Bottom water temperature	0.135
Surface sediment porosity	0.105
**Results**	
Coefficient of determination	0.510
Global Mg flux (Tmol y^{-1})	1.06

Supplementary Table 2. Random forest regression parameters and results.

Model parameters	
Data normalized	yes
Intercept used	yes
**Feature importances**	
Sedimentation rate	2.86E-03
Surface sediment porosity	-1.04E-03
Bottom water temperature	2.02E-04
Water depth	-8.12E-05
Surface ocean productivity	3.20E-04
**Results**	
Coefficient of determination	0.186
Global Mg flux (Tmol y^{-1})	1.27

Supplementary Table 3. Multiple linear regression parameters and results.

Leg/Expedition	Site	Hole	Core	Type	Section	Depth (mbsf)	δ^{26}Mg (‰)	δ^{26}Mg 2σ (‰)	δ^{25}Mg (‰)	δ^{25}Mg 2σ (‰)
170	1039	B	1	H	1	1.45	-0.86	0.04	-0.40	0.04
170	1039	B	4	H	5	28.40	-0.93	0.04	-0.45	0.04
170	1039	B	8	H	4	64.90	-1.00	0.04	-0.50	0.04
170	1039	B	12	X	3	101.35	-1.07	0.04	-0.55	0.04
170	1039	B	16	X	3	136.55	-1.00	0.04	-0.51	0.04
170	1039	B	21	X	5	187.55	-0.92	0.04	-0.46	0.04
170	1039	B	29	X	3	261.75	-0.94	0.04	-0.48	0.04
170	1039	B	37	X	5	341.70	-0.88	0.04	-0.47	0.04
170	1039	C	7	R	1	421.65	-0.77	0.04	-0.38	0.04
170	1040	B	2	H	2	8.35	-0.86	0.04	-0.45	0.04
170	1040	B	20	X	2	164.1	-0.43	0.04	-0.22	0.04
170	1040	C	28	R	3	423.35	-0.99	0.04	-0.52	0.04
170	1040	C	45	R	2	585.5	-0.64	0.04	-0.35	0.04
334	U1378	B	1	H	1	1.51	-0.81	0.06	-0.41	0.04
334	U1378	B	3	H	5	22.15	-0.68	0.06	-0.33	0.04
334	U1378	B	5	H	2	36.66	0.05	0.06	0.04	0.04
334	U1378	B	10	H	2	84.18	-0.38	0.06	-0.18	0.04
334	U1378	B	19	X	5	148.62	-0.23	0.06	-0.11	0.04
334	U1378	B	29	X	3	236.65	-0.39	0.06	-0.20	0.04
334	U1378	B	41	X	5	340.88	-0.99	0.06	-0.52	0.04
334	U1378	B	56	X	2	458.40	-1.12	0.06	-0.55	0.04
334	U1378	B	60	X	4	499.90	-0.96	0.06	-0.51	0.04
334	U1378	B	62	X	1	510.37	-1.05	0.05	-0.56	0.05
344	U1380	C	13	R	6	551.75	-1.29	0.05	-0.72	0.05
344	U1414	A	1	H	1	0.56	-0.92	0.05	-0.50	0.05
344	U1414	A	3	H	2	14.01	-0.78	0.05	-0.41	0.05
344	U1414	A	5	H	5	37.12	-0.83	0.05	-0.42	0.05
344	U1414	A	12	H	5	104.06	-0.82	0.05	-0.44	0.05
344	U1414	A	22	H	6	198.88	-0.55	0.05	-0.28	0.05
344	U1414	A	28	X	4	252.93	-0.09	0.05	-0.05	0.05
344	U1414	A	32	X	2	288.82	0.08	0.05	0.03	0.05
344	U1414	A	36	R	2	314.01	-0.07	0.05	-0.04	0.05
344	U1414	A	38	R	2	328.39	-0.14	0.05	-0.06	0.05
344	U1414	A	39	R	1	336.67	0.38	0.05	0.19	0.05
315	C0002	B	1	R	2	476.58	-0.75	0.05	-0.40	0.03
315	C0002	B	11	R	3	564.47	-0.92	0.05	-0.48	0.03
315	C0002	B	19	R	3	640.52	-0.92	0.05	-0.49	0.03
315	C0002	B	27	R	2	714.27	-1.27	0.05	-0.65	0.03
315	C0002	B	37	R	2	807.12	-1.21	0.05	-0.64	0.03
315	C0002	B	48	R	5	915.45	-1.36	0.05	-0.72	0.03
315	C0002	D	1	H	2	1.52	-0.78	0.05	-0.38	0.03
315	C0002	D	2	H	3	8.64	-0.65	0.05	-0.35	0.03
315	C0002	D	3	H	4	19.28	-0.41	0.05	-0.23	0.03
315	C0002	D	5	H	4	38.33	-0.35	0.05	-0.18	0.03
315	C0002	D	10	H	4	85.87	-0.21	0.05	-0.10	0.03
315	C0002	D	16	H	6	157.09	0.05	0.05	0.01	0.03
NGHP01	NGHP18	A	1	H	1	1.40	-0.83	0.05	-0.45	0.05
NGHP01	NGHP18	A	2	H	3	11.80	-0.84	0.05	-0.45	0.05
NGHP01	NGHP18	A	2	H	6	16.30	-0.83	0.05	-0.45	0.05
NGHP01	NGHP18	A	4	H	3	30.80	-0.75	0.05	-0.40	0.05
NGHP01	NGHP18	A	6	H	2	48.17	-0.53	0.05	-0.28	0.05
NGHP01	NGHP18	A	7	H	5	61.56	-0.44	0.05	-0.22	0.05
NGHP01	NGHP18	A	9	H	2	80.75	-0.48	0.05	-0.26	0.05
NGHP01	NGHP18	A	11	H	3	101.20	-0.50	0.05	-0.28	0.04
NGHP01	NGHP18	A	16	X	6	152.65	-0.60	0.05	-0.28	0.05

Supplementary Table 4. Pore water magnesium isotope data. All δ^{26}Mg and δ^{25}Mg data are relative to the DSM3 standard. NGHP01 refers to the Indian National Gas Hydrate Program Expedition 01.
